# The Ways to Earn Money in High Risk Addicts in Drug Court Treatment-DCT Center in Zahedan TaL-e-Siah

**DOI:** 10.5812/ijhrba.12531

**Published:** 2013-06-26

**Authors:** Mostafa Dah Mardei, Saeedeh Olyaee

**Affiliations:** 1 Department of Medicine, Zahedan University of Medical Sciences, Zahedan, IR Iran

**Keywords:** Addiction, Risk, Behavior

Dear Editor,

Drug dependence is a chronic and often recurrent disorder which is associated with many problems such as AIDS, hepatitis, etc. This disorder requires treatment management over time. Methadone maintenance therapy is one addiction treatment method which prevents not only the recurrence of drug dependence, but also it improves mental and physical status of the patient (1). Addiction compulsive treatment is one treatment measure which is used to treat addicts (2). The application of this therapy scientifically began in the world in the middle of 1980s (3). In our country, for the first time in 2007, law enforcement and health care systems jointly developed a protocol to provide a treatment measure for this disorder and reduce complications associated with compulsory treatment (4). These centers are established in many provinces. They were launched in Zahedan in the TaL-e-Siah area in February 2012. First, the addicts were arrested by police force. Then, they were transferred to health care center for methadone maintenance therapy after they were screened by police force. One criterion justifying entrance of addicts to these centers (the reason behind transferring the addicts to health care centers) was that these patients were homelessness and had no permanent and suitable jobs for living affairs. Due to lack of jobs, these patients tend to various ways to get drug.

A few numbers of researches have been done in this field in these centers since this therapy is still new and were not used for treatment much. For example, the research result of compulsory treatment center of Shafagh Tehran showed that only 17.7% of addicts had permanent jobs or were retired while 82.3% of addicts had no jobs or had temporary or illegal jobs (2). This study was designed and conducted with the purpose of examining ways to earn money in addicts who were involuntary admitted to compulsory treatment center of Zahedan TaL-e-Siah.

This was a cross-sectional study which type was descriptive - analytical. The sample was selected from all addicted male individuals who were arrested by police force. Among these arrested addicts, 100 individuals were selected and transferred to Zahedan TaL-e-Siah center to be treated by methadone maintenance therapy. Data collection was done by a questionnaire which consisted of 19 questions. These questions included demographic data as well as information regarding ways to earn money in addicts as follows: beggary, theft, junk seeking, extortion, family, drug sales, etc. Research data was also collected from interviews which questioner had performed and data analysis by SPSS 19 software. The results showed that mean age of these patients was 32 years old in which 49% of them had education level under elementary school, 34% of them had education level under diploma, 17 % of them had diploma and education level higher than diploma, 40 % of them had a history of suicide and self-mutilation, 68 % of them had a history of imprisonment, 55 % of them were single and 45 % of them were married. Sixty-two percent of these addicts had stated that their source of income was their families; however, they had taken money from their families by force. Other sources of incomes of these patients were as follows: 24% theft, 22% junk seeking, 20% drug sales, 17% proletarian, 14% temporary job, 8% Driving, 5% extortion, 4% dismissed employee of the government, 3% contraband. According to the results of the research, and higher percent of unemployment and criminal records of the addicts, it can be said that drug consumption has probably a direct relationship with unemployment and crime commitment. In other words, if unemployment among addicts increases, drug consumption and tendency to fake incomes will increase. The addicts inevitably tend to commit crime because no employer would willingly give them a job. Therefore, the addict tends to theft, junk seeking, drug sale, beggary, etc. in order to buy drug for themselves. As a result, this damaged cycle will repeat again and again. Moreover, high percent of suicide and self-mutilation background represent despair and depression and borderline personality disorder and other psychiatric disorders in these addicts. This issue needs immediate treatment interventions. One purpose of establishing compulsive drug treatment centers is to teach some profession to the addicts in various fields through vocational training and employment organization. If this comes off, hopefully fake jobs and crime commitment would reduce and as a result drug demand would decrease.
